# The resilience of the inner ear—vestibular and audiometric impact of transmastoid semicircular canal plugging

**DOI:** 10.1007/s00415-021-10693-5

**Published:** 2021-08-10

**Authors:** Joost J. A. Stultiens, Nils Guinand, Vincent Van Rompaey, Angélica Pérez Fornos, Henricus P. M. Kunst, Hermanus Kingma, Raymond van de Berg

**Affiliations:** 1 Department of Otorhinolaryngology & Head and Neck Surgery, Faculty of Health Medicine and Life Sciences, School for Mental Health and Neuroscience, Maastricht University Medical Center, Maastricht, The Netherlands; 2 Division of Otorhinolaryngology and Head-and-Neck Surgery, Department of Clinical Neurosciences, Geneva University Hospitals, Geneva, Switzerland; 3 Department of Otorhinolaryngology and Head & Neck Surgery, Faculty of Medicine and Health Sciences, Antwerp University Hospital, University of Antwerp, Antwerp, Belgium

**Keywords:** Semicircular canals, Plugging, Occlusion, Vestibular function, Hearing, Vestibular implant

## Abstract

**Background:**

Certain cases of superior semicircular canal dehiscence or benign paroxysmal positional vertigo can be treated by plugging of the affected semicircular canal. However, the extent of the impact on vestibular function and hearing during postoperative follow-up is not known.

**Objective:**

To evaluate the evolution of vestibular function and hearing after plugging of a semicircular canal.

**Methods:**

Six patients underwent testing before and 1 week, 2 months, and 6 months after plugging of the superior or posterior semicircular canal. Testing included caloric irrigation test, video Head Impulse Test (vHIT), cervical and ocular Vestibular Evoked Myogenic Potentials (VEMPs) and audiometry.

**Results:**

Initially, ipsilateral caloric response decreased in all patients and vHIT vestibulo-ocular reflex (VOR) gain of each ipsilateral semicircular canal decreased in 4/6 patients. In 4/6 patients, postoperative caloric response recovered to > 60% of the preoperative value. In 5/6 patients, vHIT VOR gain was restored to > 85% of the preoperative value for both ipsilateral non-plugged semicircular canals. In the plugged semicircular canal, this gain decreased in 4/5 patients and recovered to > 50% of the preoperative value. Four patients preserved cervical and ocular VEMP responses. Bone conduction hearing deteriorated in 3/6 patients, but recovered within 6 months postoperatively, although one patient had a persistent loss of 15 dB at 8 kHz.

**Conclusion:**

Plugging of a semicircular canal can affect both vestibular function and hearing. After initial deterioration, most patients show recovery during follow-up. However, a vestibular function loss or high-frequency hearing loss can persist. This stresses the importance of adequate counseling of patients considering plugging of a semicircular canal.

**Supplementary Information:**

The online version contains supplementary material available at 10.1007/s00415-021-10693-5.

## Introduction

Few vestibular disorders require surgery to relief complaints. Patients suffering from vestibular pathologies are often, depending on the pathology, offered conservative treatment, such as repositioning maneuvers [1], rehabilitation therapy [2, 3] or medication [4]. For many vestibular disorders no cure is available yet [5, 6]. However, in certain patients disabling complaints are not sufficiently improved by conservative therapy and the underlying pathophysiology might demand surgical treatment. Symptomatic superior canal dehiscence syndrome (SCDS) is one of these vestibular disorders that can cause a serious reduction in quality of life [7]. The symptoms are caused by an absence of bone covering the superior semicircular canal. Patients suffering from disabling vestibular and/or auditory symptoms may benefit from superior semicircular canal surgery, to eliminate the so-called ‘third mobile window’, as this may lead to a change in pressure transmission in the inner ear [8]. Plugging of this canal seems to be the most favorable surgical technique [9]. Surgical plugging of a semicircular canal can also be used to treat certain intractable cases of benign paroxysmal positional vertigo (BPPV). This disorder is characterized by recurrent attacks of positional vertigo or dizziness, provoked by lying down or turning over in supine position. It is believed to be caused by dislodged otoconia that end up in a semicircular canal resulting in stimulation of the canal upon postural changes. The posterior semicircular canal is most frequently affected [10]. Most cases can be treated successfully with repositioning maneuvers, but patients with disabling and intractable BPPV may benefit from surgical plugging of the affected semicircular canal to alleviate symptoms [11].

The abovementioned surgeries seem to be helpful to relieve vestibular symptoms, but also involve opening of the inner ear. Consequently, there is a risk of inducing sensorineural hearing loss and/or loss of vestibular function. Previous studies have reported cases of transient hearing loss [12], persistent (high-frequency) sensorineural hearing loss [12–14], a decrease in vestibulo-ocular reflex (VOR) gain of all ipsilateral semicircular canals [15, 16], or an increase in semicircular canal paresis, up to caloric areflexia [17–19]. Unfortunately, structured data on the occurrence of these side effects is lacking, especially regarding objective vestibular function [11, 20]. Furthermore, since many studies use a single moment of postoperative evaluation and the follow-up interval differs between studies, assessment of the evolution of inner ear function over time is impeded [11, 20].

Currently, the feasibility of vestibular implantation is also being investigated to treat disabling vestibular hypofunction [21–23]. This technology utilizes electrodes implanted either within the semicircular canals in the vicinity of the ampullary nerve fibers (intralabyrinthine approach) or directly onto these nerves outside the labyrinth (extralabyrinthine approach) [24]. Consequently, when using the intralabyrinthine approach, the electrode also partially occludes the semicircular canal and may, therefore, interfere similarly with inner ear function. Previous research has shown that this procedure may affect both auditory and residual vestibular function [21, 25], but the extent of its impact is unknown. However, this can be very relevant for patients with vestibular hypofunction that still have good hearing and also in the future for patients with residual vestibular function who may become vestibular implant candidates.

The aim of this study was to evaluate the evolution of vestibular function and hearing after plugging of a semicircular canal. This could aid the decision-making process and counseling of patients that may undergo semicircular canal plugging. Furthermore, this information may be helpful in the development and clinical implementation of other procedures involving semicircular canal surgery, such as vestibular implantation.

## Materials and methods

### Patients and study protocol

A prospective cohort study was performed. Patients scheduled to undergo plugging of a semicircular canal at a tertiary referral center were selected. This included plugging of the superior semicircular canal for disabling SCDS and plugging of the posterior semicircular canal for intractable BPPV. All patients underwent audiometry and vestibular testing preoperatively, and at 1 week, 2 months and 6 months postoperatively. Besides, the subjective effect was assessed during these follow-up visits. In addition, a CT scan was performed for clinical purpose 6 months postoperatively.

### Surgery

Surgery was performed by three different neuro-otologists (RvdB, VVR, and HKu) at the Maastricht University Medical Center+. The surgical procedures for plugging of the superior and posterior semicircular canal were similar. First, a cortical mastoidectomy was performed and the bony part of the affected semicircular canal was exposed. In the SCDS cases, the semicircular canal was skeletonized proximally and distally of the dehiscence. At each of these locations, a fenestration was made. Subsequently, fascia or fat was inserted through both fenestrations in the direction of the vestibule to create two ‘plugs’. Then, the remaining part of covering bone between the fenestrations was removed to be able to identify the dehiscence and the remnant was covered with a mix of bone paté and fibrin glue (Tisseel, Baxter, Deerfeeld, Illinois, USA). In the BPPV cases, the canal was skeletonized for a length of 4–6 mm and one fenestration of approximately 3–4 mm was created. Then plugs of fascia or fat were inserted both proximally and distally of the fenestration. Between these plugs, the canal was closed with a mix of bone paté and fibrin glue.

### Vestibular and audiometric testing

Vestibular assessment consisted of the caloric test, video Head Impulse Test (vHIT) and cervical and ocular vestibular evoked myogenic potentials (cVEMP and oVEMP) preoperatively and at 1 week, 2 months and 6 months postoperatively (Table 1). The following oculomotor tests were performed to rule out central lesions: smooth pursuit, saccades, optokinetic nystagmus, spontaneous nystagmus and gaze nystagmus testing.

**Table 1:** Performed examinations at the different evaluation intervals

	Preoperative	Postoperative		
		1 week	2 months	6 months
Caloric test	✔		✔	✔
vHIT	✔	✔	✔	✔
oVEMP	✔		✔	
cVEMP	✔		✔	
Audiometry	✔	✔	✔	✔
CT scan	✔			✔

Bithermal caloric testing (30 °C and 44 °C) was performed in a completely dark room using water irrigations (Variotherm plus, Atmos Medizin Technik GmbH, Lenzkirch, Germany). Eye movement calibration was performed before each irrigation. Eye movements were recorded using electronystagmography (Kingslab 1.8.1, Maastricht University, Maastricht, The Netherlands).

The vHIT was performed, while the patient sat in a chair and focused on a dot at 1.5 m on the opposite wall. After calibration, the examiner performed fast angular head movements in the planes of the semicircular canals (right horizontal–left horizontal, right superior–left posterior and left superior–right posterior). Video goggles with a motion tracking sensor (ICS Impulse, GN Otometrics, Taastrup, Denmark) recorded eye and head movements. This yielded mean VOR gains of all semicircular canals, calculated by the vHIT system as the ratio of the area under the curve of eye velocity and of head velocity (from 60 ms before peak head acceleration to the last value of 0°/s as the head returns to rest).

cVEMP thresholds were determined by measuring sternocleidomastoid muscle inhibition using electromyography (Neuro-Audio, Difra Instrumentation, Eupen, Belgium), while providing tone bursts of 500 Hz at repetition rates of 5 Hz or 13 Hz through inserted earphones (ipsilaterally). The stimulus contained a rise and a fall time of each one cycle (2 ms). A staircase approach with steps of 5 dB SPL was performed, starting at 120 dB SPL for normal hearing patients or maximally 130 dB SPL for patients with hearing loss. A minimum of two hundred electromyography traces with a mean rectified voltage of minimally 65 µV and maximally 205 µV were included. Baseline muscle tension was ensured using a visual feedback system. If there was an air–bone gap present at 0.5 kHz in the pertaining audiogram, this air–bone gap was subtracted from the cVEMP threshold to correct for possible conductive hearing loss influencing the results. The procedure was similar for determining oVEMP thresholds, in which inferior oblique muscle activation was measured while stimulating the contralateral ear, using a minimum of 300 electromyography traces.

Pure-tone audiometry was performed according to clinical standards in a sound-treated room preoperatively and at all postoperative follow-up intervals. Bone conduction and air conduction thresholds were determined for both ears at 0.25, 0.5, 1, 2, 4 and 8 kHz. Pure tone averages of the bone conduction thresholds at 1, 2 and 4 kHz were calculated (PTA_1,2,4_).

### Analysis

The vestibular data consisted of summed maximum slow phase eye velocity (warm and cold irrigation) on each side, mean VOR gain (vHIT) and stimulation thresholds (dB for cVEMP and for oVEMP). Audiometric thresholds for the frequencies 1, 2 and 4 kHz were averaged to calculate the pure tone average (PTA_1,2,4_). Evolution of individual values was assessed. Due to the small sample size, only descriptive statistics were used. An increase of ≥ 10 dB in bone conduction PTA_1,2,4_ was assessed to indicate sensorineural hearing loss, an increase of ≥ 10 dB at the individual air conduction threshold at 8 kHz was assessed to reflect high-frequency hearing loss. For the latter, air conduction thresholds were chosen to limit bias by ‘supranormal’ or third-window induced improved bone conduction thresholds preoperatively (see “Discussion”). Furthermore, vestibular data were normalized to the preoperative value to assess individual proportional decline and restoration. The lateral semicircular canal was evaluated as an indicator of (residual) function of the ipsilateral vestibular system. The extent of the plugging on the CT images was assessed by a neuroradiologist.

### Ethics

The procedures in this investigation were in accordance with legislation and ethical standards on human experimentation in the Netherlands. Approval was sought from an ethical committee. The study was approved by the institutional ethics committee “METC azM/UM” (METC 2019-1359).

## Results

### Patient characteristics

Six patients were included and all completed follow-up after surgery. This included four patients suffering from SCDS and two patients suffering from posterior semicircular canal BPPV. Median age at surgery was 51 years (range 39–64 years). Patient characteristics are presented in Table 2. Baseline vestibular and hearing function data are presented in Table 3. Data from the contralateral ear is included as supplementary material (Supplementary Information, containing Tables S1-S4).

**Table 2:** Patient characteristics

#	Condition	Laterality	Age	Sex
1	SCDS	Left	52	Female
2	SCDS	Right	61	Male
3	SCDS	Left	50	Male
4	SCDS	Left	43	Male
5	BPPV	Right	64	Female
6	BPPV	Left	39	Male

SCDS: superior canal dehiscence syndrome, BPPV: benign paroxysmal positional vertigo

**Table 3:** Baseline patient data of the ipsilateral ear

#	Plugged SCC	BC PTA_1,2,4_ (dB HL)	AC PTA_1,2,4_ (dB HL)	Caloric sMSPV (°/s)	LSCC VOR gain	nPvSCC VOR gain	PvSCC VOR gain	cVEMP threshold (dB SPL)	oVEMP threshold (dB SPL)
1	Superior SCC	20	25	45	0.84	0.72	0.62	90	85
2	Superior SCC	65	72	23	0.91	0.64	− 0.04	95	100
3	Superior SCC	27	28	24	0.85	1.09	0.47	75	70
4	Superior SCC	15	15	49	0.89	0.86	0.91	100	95
5	Posterior SCC	22	27	65	0.85	0.29	0.97	110	125
6	Posterior SCC	22	22	8	0.91	0.67	0.53	120	115
$\tilde{x}$	Median	22	26	35	0.87	0.70	0.58	98	98

SCC: semicircular canal; BC PTA_1,2,4_/AC PTA_1,2,4_: pure tone average of the thresholds at 1, 2 and 4 kHz stimulation for bone conduction and air conduction, respectively; sMSPV: summed maximum slow-phase eye velocity of bithermal caloric testing; LSCC, nPvSCC, PvSCC VOR gain: vestibulo-ocular reflex gain of the video head impulse test of the lateral, non-plugged vertical, and plugged vertical semicircular canal, respectively; cVEMP, oVEMP: thresholds of cervical and ocular Vestibular Evoked Myogenic Potentials, respectively

### Vestibular findings

**Fig. 1:**
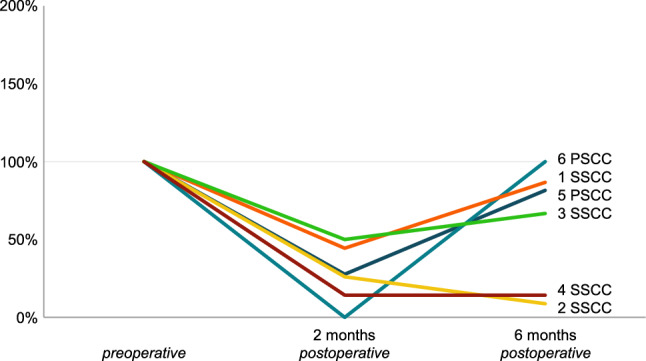
Caloric test results (summed maximum slow phase velocity of the eye) of the operated side of six patients before and after surgical plugging of a vertical semicircular canal, normalized to the preoperative value (100%). Each patient is color coded and presented as a number combined with the plugged semicircular canal (SSCC or PSCC for the superior or posterior semicircular canal, respectively)

**Fig. 2:**
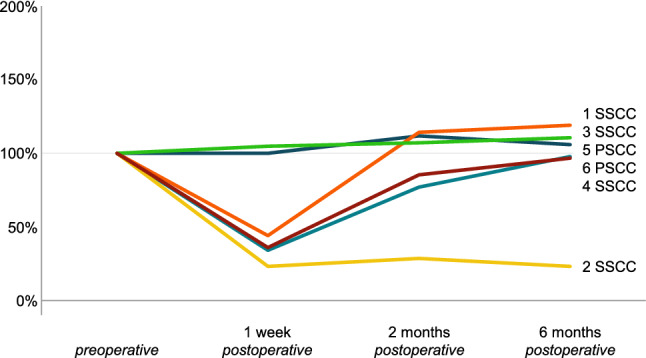
Video head impulse test vestibulo-ocular reflex gain results for the lateral semicircular canal of the operated side of six patients before and after surgical plugging of a vertical semicircular canal, normalized to the preoperative value (100%). Each patient is color coded and presented as a number combined with the plugged semicircular canal (SSCC or PSCC for the superior or posterior semicircular canal, respectively)

**Fig. 3:**
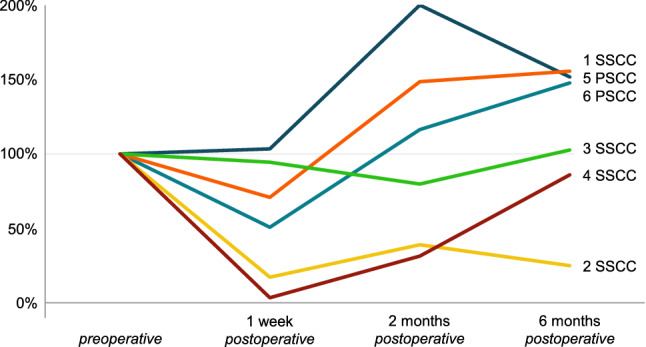
Video head impulse test vestibulo-ocular reflex gain results for the non-plugged vertical (i.e., posterior or superior) semicircular canal of the operated side of six patients before and after surgical plugging of a vertical semicircular canal, normalized to the preoperative value (100%). Each patient is color coded and presented as a number combined with the plugged semicircular canal (SSCC or PSCC for the superior or posterior semicircular canal, respectively)

**Fig. 4:**
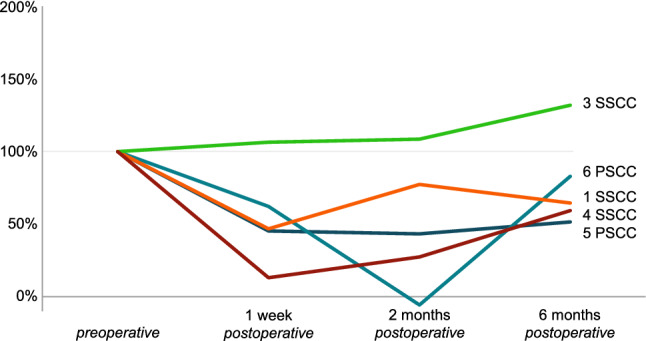
Video Head Impulse Test (vHIT) vestibulo-ocular reflex (VOR) gain results for the plugged (i.e., posterior or superior) semicircular canal of the operated side of five patients before and after surgical plugging of a vertical semicircular canal, normalized to the preoperative value as 100%. Each patient is presented as a number combined with the plugged semicircular canal (SSCC or PSCC for the superior or posterior semicircular canal, respectively). Data from patient #2 is not shown, because he had an absent vHIT VOR gain preoperatively (− 0.04), with a VOR gain of 0.30–0.33 during the follow-up visits, which would hamper visualization of these normalized data

Vestibular results of the plugged semicircular canal and the total ipsilateral vestibular function are presented in Figs. 1, 2, 3 and 4 and Tables 4, 5 and 6. Plugged semicircular canal function as well as the function of the other ipsilateral vestibular sensors showed impaired test results postoperatively. An initial decline in caloric test response was present in all patients (Fig. 1), while vHIT VOR gain of all ipsilateral semicircular canals declined in four patients (Figs. 2 and 3). In four out of six patients, caloric response recovered to > 60% of the preoperative value during 6 month follow-up (Figs. 1, 2 and 3) and vHIT VOR gain was restored to ≥ 98% of the preoperative value for both ipsilateral non-plugged semicircular canals (Figs. 2 and 3). One of the two patients without recovery on caloric test results (patient #4) still showed recovery to > 85% on vHIT VOR gain of both ipsilateral non-plugged semicircular canals (Figs. 1, 2 and 3).

**Table 4:** Objective outcome data of the ipsilateral ear at 1 week postoperative follow-up

#	Plugged SCC	BC PTA_1,2,4_ (dB HL)	AC PTA_1,2,4_ (dB HL)	LSCC VOR gain	nPvSCC VOR gain	PvSCC VOR gain
1	Superior SCC	45	48	0.37	0.51	0.29
2	Superior SCC	58	70	0.21	0.11	0.32
3	Superior SCC	33	60	0.89	1.03	0.50
4	Superior SCC	28	40	0.32	0.03	0.12
5	Posterior SCC	20	27	0.85	0.30	0.44
6	Posterior SCC	35	40	0.31	0.34	0.33
$\tilde{x}$	Median	34	44	0.35	0.32	0.33

Legends an analogous to Table 3.

**Table 5:** Objective outcome data of the ipsilateral ear at 2 month postoperative follow-up

#	Plugged SCC	BC PTA_1,2,4_ (dB HL)	AC PTA_1,2,4_ (dB HL)	Caloric sMSPV (°/s)	LSCC VOR gain	nPvSCC VOR gain	PvSCC VOR gain	cVEMP threshold (dB SPL)	oVEMP threshold (dB SPL)
1	Superior SCC	28	32	20	0.96	1.07	0.48	x	115
2	Superior SCC	68	72	6	0.26	0.25	0.33	110	125
3	Superior SCC	22	30	12	0.91	0.87	0.51	100	105
4	Superior SCC	15	18	7	0.76	0.27	0.25	85	110
5	Posterior SCC	25	30	18	0.95	0.58	0.42	130	130
6	Posterior SCC	25	30	0	0.70	0.78	− 0.03	x	x
$\tilde{x}$	Median	25	30	10	0.84	0.68	0.38		

*x*: No response obtained at the highest stimulation level

Further legends analogous to Table 3

**Table 6:** Objective outcome data of the ipsilateral ear at 6 month postoperative follow-up

#	Plugged SCC	BC PTA_1,2,4_ (dB HL)	AC PTA_1,2,4_ (dB HL)	Caloric sMSPV (°/s)	LSCC VOR gain	nPvSCC VOR gain	PvSCC VOR gain
1	Superior SCC	17	30	39	1.00	1.12	0.40
2	Superior SCC	68	73	2	0.21	0.16	0.30
3	Superior SCC	25	25	16	0.94	1.12	0.62
4	Superior SCC	15	18	7	0.86	0.74	0.54
5	Posterior SCC	28	35	53	0.90	0.44	0.50
6	Posterior SCC	25	30	8	0.89	0.99	0.44
$\tilde{x}$	*Median*	*25*	*30*	*12*	*0.90*	*0.87*	*0.47*

Legends analogous to Table 3

Plugged semicircular canal function, as measured by vHIT, decreased initially in four of the five patients with a present preoperative vHIT VOR gain (i.e., > 0; Fig. 4). Recovery during follow-up varied between patients. At 6 months postoperatively, vHIT VOR gain of this semicircular canal recovered to ≥ 52% of the preoperative value for all patients.

VEMP testing showed presence of both ocular and cervical VEMPs before plugging in all patients. Thresholds increased in most patients, one patient (patient #4) had a decreased cVEMP threshold postoperatively. Four patients retained both cVEMP and oVEMP responsiveness. One patient who underwent plugging of the posterior semicircular canal (patient #6) did not preserve cVEMP or oVEMP thresholds. Preoperatively, his thresholds were 120 dB for cVEMP and 115 dB for oVEMP. One patient who underwent plugging of the superior semicircular canal (patient #1) had an absent cVEMP response postoperatively (90 dB preoperatively).

Dix-Hallpike and supine roll test were negative postoperatively in all patients.

### Audiometric findings

**Fig. 5:**
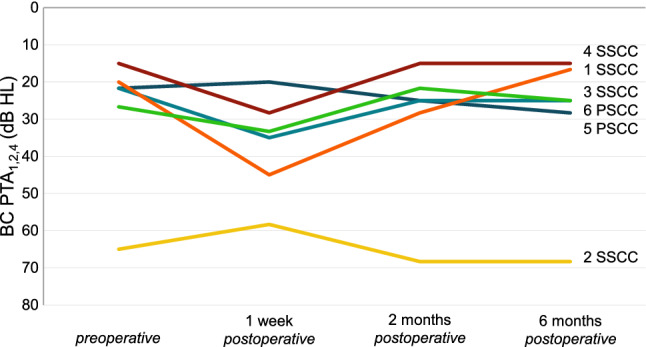
Bone conduction thresholds of the pure tone average of the frequencies 1, 2 and 4 kHz (BC PTA_1,2,4_) of the operated side of six patients before and after surgical plugging of a vertical semicircular canal. Each patient is presented as a number combined with the plugged semicircular canal (SSCC or PSCC for the superior or posterior semicircular canal, respectively)

At 1 week follow-up, bone conduction PTA_1,2,4_ was decreased 10 dB or more compared to preoperative testing in 3/6 patients (patients #1, #4 and #6) and the 8 kHz stimulation threshold had deteriorated 10 dB or more in 3/6 patients (patients #1, #3 and #4). The bone conduction PTA_1,2,4_ of the former three patients was restored to less than 10 dB of the preoperative value within 2 months (Fig. 5). One patient (patient #1) remained with a deterioration of 15 dB at 8 kHz after 6 months.

### Imaging

In five patients an occluding plug was visible on the CT images 6 months postoperatively, while in one patient (patient #5), the extent of the plugging could not be determined.

### Subjective outcomes

The four patients with SCDS had postoperative resolution of disabling autophony and pulsatile tinnitus. The two patients that suffered from BPPV did not experience episodes of BPPV during 6 month postoperative follow-up. All patients except patient #2 had postoperative complaints of dizziness or imbalance, especially with fast head movements. These complaints improved partially (patients #1, 3, 4, 5) or fully (patient #6), during follow-up. All patients indicated to be satisfied with the postoperative result.

## Discussion

This study presents systematically assessed structured vestibular and audiometric follow-up results after plugging of a semicircular canal in six vestibular patients. These results indicate that plugging of one semicircular canal can impact the whole ipsilateral vestibular system and hearing. Caloric response was initially affected in all patients. Several patients showed substantial recovery on one or more vestibular tests during 6 month follow-up. One patient remained with a substantially impaired caloric test and vHIT response. Likewise, an initial hearing deterioration that was present in some patients resolved completely, except for one patient who remained with a deteriorated hearing threshold of 15 dB at 8 kHz.

### Importance of timing of vestibular testing

The present results emphasize the importance of a systematic follow-up to assess the impact of plugging of a semicircular canal on inner ear function. Systematic objective vestibular results are lacking and reported outcomes vary between studies [11, 15, 16, 18–20, 26–28]. Mantokoudis et al. evaluated three patients up to 1–4 months after plugging of the superior semicircular canal through a middle cranial fossa approach and showed recovery of lateral semicircular canal gain in 3/3 patients, while 1/3 showed recovery of posterior semicircular canal gain [15]. Other studies only used a single moment for postoperative vestibular evaluation, and the timing of postoperative assessment was often within 2 months, or was not described. The high dependency of the vestibular outcomes on the moment of postoperative evaluation, together with the observed inter-individual variation, can thus explain variation in the reported impact of plugging of a semicircular canal on vestibular function. Therefore, the timing of follow-up assessment is essential for interpretation of results and should be taken into account when designing future studies.

### Non-plugged semicircular canal function

There was a discrepancy between caloric test and vHIT results. Caloric response was initially affected in all patients, while vHIT VOR gain remained present in the non-plugged semicircular canals in some patients. In addition, two out of six patients did not show recovery on the caloric test, while one of these patients did recover substantially on vHIT VOR gain of both non-plugged semicircular canals (patient #4). This discrepancy may be due to a selective damage to either the low-frequency sensitivity (reflected in caloric test) or high-frequency sensitivity (reflected in vHIT) of the vestibular system. Caloric response may also be partially decreased by the cortical mastoidectomy that is part of the surgical procedure, since this may lead to a decrease in thermal conduction properties of the mastoid, which may improve again after possible formation of connective tissue in the mastoid. Nevertheless, it is very likely that a temporary low frequency vestibular hypofunction occurred, since other studies showed that several patients maintain similar caloric response within 2 months after extensive mastoid drilling, e.g., in cochlear implant surgery [29, 30], while in the present study, caloric response of all six patients deteriorated. Furthermore, it was found that some patients even had improved vHIT VOR gain values at 6 month follow-up compared to the preoperative value, mainly in the non-plugged vertical semicircular canal. Measurement of vHIT VOR gain in the vertical semicircular canals generally contains larger variations compared to this measurement in the lateral semicircular canals. Head rotation speed is usually lower and artifacts occur more often. However, whether the observed improvement of vHIT VOR gain is caused by a variability in measurements, measurement artifacts or a modification of the biomechanical properties of the inner ear, could not be determined by this study.

### Plugged semicircular canal function

Interestingly, in some patients the vHIT VOR gain of the plugged semicircular canal also showed partial recovery, even though the occlusion created a new end of the canal, clearly visible on CT. Four out of five patients with a present preoperative VOR gain in the plugged semicircular canal, showed an initial decrease in this response after plugging, which is in line with previous findings [15, 16]. Since very small deflections of the cupula can already cause depolarization of the vestibular nerve [31], it can be hypothesized that even in a plugged semicircular canal, high-frequency movements may still initiate a vestibular response and, therefore, a VOR. All patients showed a long-term recovery that was similar to the recovery of the other semicircular canals. Taking this into account, the initial decline and sequential improvement of the plugged semicircular canal function, might most likely be attributed to two factors: the surgical plug itself and the (temporary) deterioration of the whole ipsilateral vestibular system after surgery.

### Vestibular evoked myogenic potentials

VEMP responses remained present in four out of six patients. This suggests that otolith responses can be preserved in certain cases. Increase in VEMP thresholds in the patients that underwent plugging of the superior semicircular canal may, at least partially, indicate recovery of the lower-than-normal preoperative VEMP threshold due to occlusion of the dehiscence. The postoperative disappearance of otolith responses in certain cases, may, therefore, be due to a preoperative bad responsiveness which could have been induced by the dehiscence (i.e., leading to a measurable threshold) and disappeared after the occlusion. Furthermore, these cases without postoperative VEMP response could have suffered from damage to the otoliths by the surgical procedure, which resulted either in a temporary hypofunction (similar to what was seen in certain caloric and vHIT responses) or in a permanent function loss.

### Auditory function

Hearing function showed similar results to the vestibular function. It was demonstrated that some patients had a significant decrease in bone conduction PTA_1,2,4_, but this recovered to less than 10 dB of the preoperative PTA_1,2,4_ within 2 months. However, it should be noted that an improved bone conduction due to the third-window mechanism may have been present preoperatively in the patients with SCDS [32]. Furthermore, one patient showed a persistent hearing loss at 8 kHz during 6 months of follow-up. This risk of a high-frequency hearing loss was reported previously [12, 13].

### Mechanisms of reduced inner ear function after plugging

The precise cause of reduced inner ear function after semicircular canal surgery is not clear. Modifications of the biomechanical properties of the inner ear may include direct leakage of perilymph, leakage of endolymph and local postoperative inflammation induced by tissue damage. During surgery, it was aimed to minimize these effects. Leakage of perilymph was intentionally limited by avoiding suctioning in the immediate vicinity of the fenestration. The effects of local inflammation were diminished by intra- and postoperatively administering corticosteroids. Individual factors leading to either deterioration or improvement could not be determined in this study.

### Imaging findings

Five patients showed a blockage of the operated semicircular canal on CT, while in one patient, the extent of canal occlusion could not be determined. Since not all occlusion material is radiopaque, a MRI should be performed to adequately assess the extent of canal occlusion. However, its clinical value is limited in cases in which symptoms are sufficiently relieved [33]. Since all patients were sufficiently relieved of symptoms, no postoperative MRI was performed in the patients included in this study.

### Clinical impact

The findings of this study can be used in counseling of patients that consider to undergo surgical plugging of a semicircular canal. Patients should be informed about the potential effect on inner ear function and its evolution. If patients opt for this procedure, the function of the contralateral vestibular system should also be taken into account, which is especially relevant in patients with bilateral superior semicircular canal dehiscence. Therefore, preoperative vestibular assessment is important for determining the side of surgical intervention as well as for counseling of the expected postoperative complaints.

Furthermore, these results can be used to estimate the effect of other semicircular canal surgeries, such as vestibular implantation. In recent years, efforts have been made to precisely implant electrodes in the semicircular canal ampullae in order to restore vestibular function in patients with bilateral vestibulopathy [24]. The implanted electrodes are relatively big in diameter compared to the small semicircular canal, and may, therefore, act similar to a plugged semicircular canal. It has been shown that hearing may endure during the surgical procedure [34]. However, the effect of intralabyrinthine vestibular implantation on both hearing and residual vestibular function has not been sufficiently investigated. It is also unclear whether or not these effects on the inner ear are comparable to the effects of cochlear implantation [35–37]. Yet, the robustness of the inner ear to semicircular canal surgery may be important for future development of the vestibular implant and its implementation. For example, forthcoming investigations may include implantation of patients with residual vestibular function, similar to current practice in cochlear implantation [36, 37]. Future research should, therefore, address the factors that influence the degree of inner ear preservation.

### Strengths and limitations

This study demonstrates a systematic prospective assessment of vestibular and audiometric function during 6 month follow-up. The evolution of the function of all semicircular canals and the otolith organs was assessed. The findings, with the lateral semicircular canal used as marker for ipsilateral vestibular function, appear representative for the impact of plugging of a semicircular canal on the ear. The results in the lateral semicircular canal of the contralateral did not change much, but there was some variation in the contralateral vertical semicircular canal, possibly because of the lower head impulse speed in this plane leading to a higher contribution of the contralateral semicircular canal (Supplementary Information). However, these canals showed a similar trend. The follow-up time seemed sufficient to evaluate functional recovery, since in most patients recovery to (almost) the preoperative value on caloric testing, vHIT VOR gain and hearing occurred within 2–6 months, although a prolonged recovery period in some patients cannot be ruled out. Delayed deterioration after 6 months could also not be evaluated, although this does not seem likely for both mechanical damage and inflammation. Due to the strict selection procedure for this surgery, only a small study sample was collected and consequently no inferential statistical analysis was performed. A larger sample size could estimate the proportions of patients that will experience a transient or a permanent loss of vestibular and/or auditory function, as well as possibly indicate which patients are more vulnerable to this deterioration. Furthermore, specific surgical steps or peri-operative procedures that might limit postoperative deterioration of inner ear function could be assessed. Nonetheless, the current evaluation gives a valuable insight in possible postoperative vestibular and auditory evolution.

## Conclusion

Plugging of a semicircular canal can affect both vestibular function and hearing. The inner ear appears quite resilient, often showing recovery after an initial deterioration. The extent of recovery and the duration of the recovery period varies between patients. Still, some patients experience persistence of impeded inner ear function. These results can be used in the decision-making process and counseling of patients considering some type of semicircular canal plugging, including intralabyrinthine vestibular implantation in the future.

## Supplementary Information

Below is the link to the electronic supplementary material.

Supplementary file1 (DOCX 33 kb)

## Data Availability

All included raw data is available upon request.
