# Supplementary material for: The resilience of the inner ear—vestibular and audiometric impact of transmastoid semicircular canal plugging
Source: J Neurol. 2021 Aug 10;269(10):5229–38. doi: 10.1007/s00415-021-10693-5 (PMC9467949; doi:10.1007/s00415-021-10693-5)
Supplement: Supplementary file 1 — Supplementary file1 (DOCX 33 kb) [file 415_2021_10693_MOESM1_ESM.docx]

**SUPPLEMENTARY INFORMATION**

**The Resilience of the Inner Ear – Vestibular and Audiometric Impact of Transmastoid Semicircular Canal Plugging**

*Journal of Neurology.*

Joost J. A. Stultiens^1^, Nils Guinand^2^, Vincent Van Rompaey^3^, Angélica Pérez Fornos^2^, Henricus P. M. Kunst^1^, Hermanus Kingma^1^, Raymond van de Berg^1^.

^1^Department of Otorhinolaryngology & Head and Neck Surgery, School for Mental Health and Neuroscience, Faculty of Health Medicine and Life Sciences, Maastricht University Medical Center, Maastricht, The Netherlands.

^2^Division of Otorhinolaryngology and Head-and-Neck Surgery, Department of Clinical Neurosciences, Geneva University Hospitals, Geneva, Switzerland.

^3^Department of Otorhinolaryngology and Head & Neck Surgery, Faculty of Medicine and Health Sciences, University of Antwerp, Antwerp University Hospital, Antwerp, Belgium.

*Corresponding author:* J.J.A. Stultiens, joost.stultiens@mumc.nl

**Table S1.** Baseline patient data of the contralateral ear

SCC: semicircular canal; BC PTA_1,2,4_, AC PTA_1,2,4_: pure tone average of the thresholds at 1, 2 and 4 kHz stimulation for bone conduction and air conduction, respectively; sMSPV: summed maximum slow-phase eye velocity of bithermal caloric stimulation; LSCC, cnPvSCC, cPvSCC VOR gain: vestibulo-ocular reflex gain of the video Head Impulse Test of the semicircular canal contralateral to the lateral, non-plugged vertical, and plugged vertical semicircular canal, respectively (e.g. if the right superior semicircular canal is plugged, cPvSCC refers to the left posterior semicircular canal); cVEMP, oVEMP: thresholds of cervical and ocular Vestibular Evoked Myogenic Potentials, respectively. x: No response obtained at the highest stimulation level.

| # | **Plugged SCC** | **BC** *PTA_1,2,4_*  *(dB HL)* | **AC**  *PTA_1,2,4_*  *(dB HL)* | **Caloric**  *sMSPV (°/s)* | **LSCC**  *VOR gain* | **cnPvSCC**  *VOR gain* | **cPvSCC**  *VOR gain* | **cVEMP**  *threshold*  *(dB SPL)* | **oVEMP**  *threshold*  *(dB SPL)* |
| --- | --- | --- | --- | --- | --- | --- | --- | --- | --- |
| 1 | Superior SCC | 17 | 20 | 44 | 0.97 | 0.83 | 0.81 | 120 | 120 |
| 2 | Superior SCC | 27 | 28 | 48 | 0.78 | 0.30 | 0.36 | 85 | 85 |
| 3 | Superior SCC | 20 | 25 | 22 | 0.94 | 1.07 | 0.94 | 120 | 110 |
| 4 | Superior SCC | 20 | 20 | 31 | 0.95 | 0.80 | 0.84 | 105 | x |
| 5 | Posterior SCC | 23 | 30 | 49 | 0.79 | 0.49 | 0.32 | 105 | 130 |
| 6 | Posterior SCC | 0 | 2 | 58 | 0.97 | 0.58 | 0.76 | 120 | 125 |
| *x̃* | *Median* | *20* | *23* | *46* | *0.95* | *0.69* | *0.79* | *113* |  |

**Table S2.** Objective outcome data of the contralateral ear at one week postoperative follow-up

| # | **Plugged SCC** | **BC** *PTA_1,2,4_*  *(dB HL)* | **AC** *PTA_1,2,4_*  *(dB HL)* | **LSCC**  *VOR gain* | **cnPvSCC**  *VOR gain* | **cPvSCC**  *VOR gain* |
| --- | --- | --- | --- | --- | --- | --- |
| 1 | Superior SCC | 15 | 18 | 0.84 | 0.72 | 0.76 |
| 2 | Superior SCC | 28 | 28 | 0.77 | 0.16 | 0.84 |
| 3 | Superior SCC | 22 | 23 | 0.94 | 1.07 | 0.90 |
| 4 | Superior SCC | 18 | 18 | 0.77 | 0.83 | 0.57 |
| 5 | Posterior SCC | 18 | 28 | 0.81 | 0.34 | 0.48 |
| 6 | Posterior SCC | 3 | 7 | 0.62 | 0.51 | 0.82 |
| *x̃* | *Median* | *18* | *21* | *0.79* | *0.62* | *0.79* |

Legends analogous to Table S1.

**Table S3.** Objective outcome data of the contralateral ear at two months postoperative follow-up

| # | **Plugged SCC** | **BC** *PTA_1,2,4_*  *(dB HL)* | **AC** *PTA_1,2,4_*  *(dB HL)* | **Caloric**  *sMSPV (°/s)* | **LSCC**  *VOR gain* | **cnPvSCC**  *VOR gain* | **cPvSCC**  *VOR gain* | **cVEMP**  *threshold*  *(dB SPL)* | **oVEMP**  *threshold*  *(dB SPL)* |
| --- | --- | --- | --- | --- | --- | --- | --- | --- | --- |
| 1 | Superior SCC | 23 | 20 | 51 | 1.02 | 0.96 | 0.77 | 120 | 125 |
| 2 | Superior SCC | 32 | 32 | 48 | 0.70 | 0.36 | 0.77 | 85 | 75 |
| 3 | Superior SCC | 22 | 27 | 20 | 0.96 | 0.97 | 0.80 | 120 | 125 |
| 4 | Superior SCC | 20 | 20 | 51 | 0.87 | 0.88 | 0.52 | 75 | 110 |
| 5 | Posterior SCC | 22 | 28 | 58 | 0.83 | 0.91 | 0.54 | 105 | 130 |
| 6 | Posterior SCC | 0 | 3 | 38 | 0.76 | 0.56 | 0.74 | 125 | 130 |
| *x̃* | *Median* | *22* | *23* | *50* | *0.85* | *0.90* | *0.76* | *113* | *125* |

Legends analogous to Table S1.

**Table S4.** Objective outcome data of the contralateral ear at six months postoperative follow-up

| # | **Plugged SCC** | **BC** *PTA_1,2,4_*  *(dB HL)* | **AC** *PTA_1,2,4_*  *(dB HL)* | **Caloric**  *sMSPV (°/s)* | **LSCC**  *VOR gain* | **cnPvSCC**  *VOR gain* | **cPvSCC**  *VOR gain* |
| --- | --- | --- | --- | --- | --- | --- | --- |
| 1 | Superior SCC | 18 | 20 | 57 | 1.01 | 1.13 | 0.77 |
| 2 | Superior SCC | 33 | 37 | 40 | 0.71 | -0.01 | 0.77 |
| 3 | Superior SCC | 23 | 27 | 30 | 1.00 | 1.12 | 0.78 |
| 4 | Superior SCC | 18 | 22 | 43 | 0.91 | 0.83 | 0.71 |
| 5 | Posterior SCC | 33 | 35 | 59 | 0.85 | 0.69 | 0.42 |
| 6 | Posterior SCC | 0 | 5 | 47 | 0.88 | 0.74 | 0.87 |
| *x̃* | *Median* | *21* | *24* | *45* | *0.90* | *0.79* | *0.77* |

Legends analogous to Table S1.
